# Infection-mediated asthma: etiology, mechanisms and treatment options, with focus on *Chlamydia pneumoniae* and macrolides

**DOI:** 10.1186/s12931-017-0584-z

**Published:** 2017-05-19

**Authors:** Wilmore C. Webley, David L. Hahn

**Affiliations:** 1 University of Massachusetts Amherst, 240 Thatcher Rd. Life Science Laboratory Building N229, Amherst, MA 01003 USA; 2 University of Wisconsin School of Medicine and Public Health, 1100 Delaplaine Court, Madison, WI 53715 USA

**Keywords:** Asthma, *Chlamydia pneumoniae*, Infection, Azithromycin, Hyperresponsive, Exacerbation, Pathogenesis

## Abstract

Asthma is a chronic respiratory disease characterized by reversible airway obstruction and airway hyperresponsiveness to non-specific bronchoconstriction agonists as the primary underlying pathophysiology. The worldwide incidence of asthma has increased dramatically in the last 40 years. According to World Health Organization (WHO) estimates, over 300 million children and adults worldwide currently suffer from this incurable disease and 255,000 die from the disease each year. It is now well accepted that asthma is a heterogeneous syndrome and many clinical subtypes have been described. Viral infections such as respiratory syncytial virus (RSV) and human rhinovirus (hRV) have been implicated in asthma exacerbation in children because of their ability to cause severe airway inflammation and wheezing. Infections with atypical bacteria also appear to play a role in the induction and exacerbation of asthma in both children and adults. Recent studies confirm the existence of an infectious asthma etiology mediated by *Chlamydia pneumoniae* (CP) and possibly by other viral, bacterial and fungal microbes. It is also likely that early-life infections with microbes such as CP could lead to alterations in the lung microbiome that significantly affect asthma risk and treatment outcomes. These infectious microbes may exacerbate the symptoms of established chronic asthma and may even contribute to the initial development of the clinical onset of the disease. It is now becoming more widely accepted that patterns of airway inflammation differ based on the trigger responsible for asthma initiation and exacerbation. Therefore, a better understanding of asthma subtypes is now being explored more aggressively, not only to decipher pathophysiologic mechanisms but also to select treatment and guide prognoses. This review will explore infection-mediated asthma with special emphasis on the protean manifestations of CP lung infection, clinical characteristics of infection-mediated asthma, mechanisms involved and antibiotic treatment outcomes.

## Background

### Incidence and etiology of childhood and adult onset asthma

Asthma incidence is highest in childhood and thereafter decreases and remains stable at ~1–3 new cases per 1000 per year throughout late adolescence and adulthood [1]. In adult populations, the prevalence of active cases of childhood-onset asthma (COA) and adult-onset asthma (AOA) are approximately equal, or favor AOA [2]. Reasons for this counterintuitive prevalence ratio include (1) the propensity for COA to remit more frequently than AOA and (2) the greater number of years of adulthood in which to accrue new cases [2]. Of relevance to clinical management and population disease burden is the wide range of asthma severities, from mild intermittent to severe persistent; the most severe 20% of cases account for 80% of health care utilization and morbidity [3]. Robust population-based data indicate that around half of adults with asthma remain sub-optimally controlled, even when treated with currently available anti-inflammatory medications, and ~15% of adults with active asthma are severely uncontrolled [4–6]. These data indicate the need for novel therapies that are effective in the most severe and treatment-resistant cases of asthma that account for the majority of morbidity, mortality and health care utilization. The emerging evidence that a wide variety of microbes are present in the lower airway and may play a role in asthma pathogenesis suggests that manipulating the airway microbiome may be a novel approach towards this goal. Studies confirm the existence of an infectious etiology mediated by *Chlamydia pneumoniae* (CP) [7] and possibly other viral [8], bacterial [9] and fungal [10] microbes. Among the various infections associated with asthma, the obligate intracellular respiratory pathogen CP is of particular interest, as it is associated with both asthma severity and treatment resistance [11–13]. Although this review focuses on CP we will discuss *Mycoplasma pneumoniae* (MP) briefly under Treatment (Section V). It is possible that microbes such as CP and MP that have been implicated in recurrent wheeze and asthma etiology may serve as cofactors for viral infections, but certainly appear to act independently in asthmatic disease. The etiology of asthma remains unknown and is almost certainly multifactorial. Many “triggers” for asthma attacks are well known (e.g., allergens, viral respiratory infections, fumes, cold air, exercise) but underlying mechanisms for why some exposed individuals develop asthma while most do not remain elusive [14]. Genetic studies have failed to locate a unique “asthma gene” and instead point towards complex multifactorial genetic and environmental factors [15]. A currently popular paradigm, the “Hygiene Hypothesis,” posits that the increased incidence of allergies (hayfever and eczema) and asthma noted in recent decades, is associated with less exposure to childhood infections and bacterial products (e.g., endotoxin). Emerging evidence supports the Hygiene Hypothesis for hayfever and eczema but not for asthma which appears instead to be related to infections throughout the life cycle [16–18]. The host lung and gut microbiome as they relate to asthma are active areas of research [19]. Yet it must be pointed out that studies of bacterial rRNA may fail to detect CP due to low copy numbers or sampling problems due to deep tissue intracellular locations for this species [20, 21].

### The human microbiome and asthma risk

An increasing number of studies have now confirmed that the host microbiome has a significant impact on the risk of asthma development. A study published in 2010 by Hilty and colleagues using 16S RNA clone-library sequencing showed that when compared with healthy controls, patients with asthma had significantly more pathogenic Proteobacteria and fewer Bacteroidetes [22]. Careful assessment of both healthy controls and asthmatic patients has confirmed the presence of bacterial communities. However, the bacterial burden was significantly greater in patients with asthma than in the healthy controls [23]. The microbial burden was even greater in asthmatics with greater bronchial reactivity upon methacholine challenge. These patients showed marked improvement in bronchial reactivity to methacholine after 6 weeks on clarithromycin. Importantly, greater bronchial reactivity also correlated with greater relative abundance of members of certain bacterial communities known to exhibit characteristics that contribute to asthma pathophysiology, including species capable of inducing nitric oxide reductase, produce sphingolipids or have the ability to metabolize steroid compounds [24, 25]. A recent study showed that 1-month old infants who had positive oropharynx cultures of *Streptococcus pneumoniae, Moraxella catarrhalis, or Haemophilus influenzae* showed increased susceptibility for development of childhood asthma [19, 26]. Another recent study concluded that the nasopharyngeal microbiome within the first year of life was a determinant for infection spread to the lower airways and predicted the severity of accompanying inflammatory symptoms, as well as risk for future asthma development. The authors showed that early asymptomatic colonization of the nasopharynx with Streptococcus was a strong asthma predictor [27]. These authors also demonstrated that antibiotic usage disrupted this asymptomatic colonization and prevented asthmatic onset [27]. These findings support the hypothesis that colonization of the developing airway by certain microbes (both viral and bacterial) can significantly alter the airway architecture and overall immune function, influencing how the airway responds to a variety of insults [28]. These findings also suggest that antimicrobial agents may represent an effective therapeutic tool with the potential to curtail both the duration and severity of asthma exacerbations initiated by a variety of microbes and exposes the limitation of the hygiene hypothesis in this regard [26]. The microbiome studies cited here have not specifically targeted CP and MP in upper airways. Studies that have specifically tested for these atypical organisms have reported positive detection [29, 30]. Intracellular detection of CP in adenoid tissue of symptomatic children was extremely common [30] and raises questions regarding a potential for CP-microbiome interactions.

### Role of viral infection on wheezing and asthma exacerbation

Infections in early life can act either as inducers of wheezing or as protectors against the development of allergic disease and asthma. Many young children have wheezing episodes associated with early-life respiratory infections. The infections most likely to be associated with these wheezing episodes include respiratory syncytial virus (RSV), human rhinovirus (hRV), human metapneumovirus, parainfluenza viruses and coronavirus [31]. The hygiene hypothesis has proposed that, for some infants, frequent early life infections may protect against asthma [17] and this certainly appears to be the case for most infants, as wheezing episodes with respiratory infections diminish as the child ages. However, for others, early-life wheezing episodes may mark the beginning of asthma. Regarding established asthma, many types of viral respiratory infections have been shown to have a significant influence. In fact, viral respiratory infections are diagnosed in 80% of episodes of asthma in both children and adults [32, 33]. The question then remains; what factors determine if a viral respiratory infection provokes the onset of chronic asthma? Factors appear to include the type of virus and the viral infectious dose as well as host susceptibility factors leading to inflammation, airway cellular infiltration with neutrophils and eosinophils or the presence of allergens in the airway and their interactions with the host immune system. If this combination of host and pathogen factors results in airway inflammation and hyperresponsiveness, the outcome could be asthma. Could CP play a key role in this complex scenario? A clue to the answer to this question was found in a secondary analysis [34] of data from a community-based pediatric viral respiratory infection study that identified viral infections in 80–85% of exacerbations [33]. One hundred and eight children with asthma symptoms completed a 13-month longitudinal study in which exacerbations were recorded, and CP PCR and CP-specific secretory IgA (CP-sIgA) antibodies were measured both during exacerbations and during asymptomatic periods. CP PCR detections were similar between the symptomatic and asymptomatic episodes (23% v 28%, respectively). Children reporting multiple exacerbations remained CP PCR positive (*P* < 0.02) suggesting chronic infection. CP-sIgA antibodies were more than seven times greater in subjects reporting four or more exacerbations compared to those who reported just one (*P* < 0.02). The authors suggested that immune responses to chronic CP infection may interact with allergic inflammation to increase asthma symptoms [34]. Notably, MP was not found to be important in this study.

### *Chlamydia pneumoniae* (CP) infections and asthma initiation and severity

Emerging evidence links CP infection with both *de novo* asthma (asthma onset during/after an acute lower respiratory tract infection in a previously non-asthmatic individual – also referred to as the “infectious asthma” syndrome) and with asthma severity [11, 12, 35, 36]. This section will review what is known about CP in asthma initiation and severity, and the multiple experimentally established mechanisms that might mediate these associations. Therapeutic implications are reviewed in Section V.

*De novo* wheezing during an acute lower respiratory tract infection is remarkably common [37]. Most of these wheezing episodes appear to resolve without chronic sequelae but sometimes chronic asthma develops. Surprisingly, clinical studies report that asthma onset after an acute respiratory illness is exceedingly common (up to 45% of adult-onset asthma cases [38]. This strong temporal association of respiratory infections and asthma onset has been confirmed in a population-based study [39]. The most reliable way to establish whether a specific respiratory pathogen can initiate asthma would be to perform large, long-term prospective microbiological and clinical cohort studies of the general (non-asthmatic) population. Such a study would be very expensive and has not yet been undertaken. A second approach would be to perform prospective studies in selected non-asthmatic patients exhibiting “risk factors” for asthma in clinical settings [40]. If the selected “risk factors” do indeed identify people at higher likelihood of developing the “infectious asthma” syndrome, this type of study might be feasible. Characteristics associated with CP/MP biomarker-positive “infectious asthma” include patients with severe, treatment-resistant asthma, exhibiting a neutrophilic airway inflammation or test PCR positive for Cp or MP. It should however, be noted that there is currently no test or set of tests that will definitively diagnose who will benefit maximally from azithromycin treatment. Factors that predict risk in non-asthmatics for developing the “infectious asthma” syndrome include a previous history of self-limited lower respiratory tract illnesses such as acute bronchitis (often with wheezing) and/or pneumonia [35, 38, 39]. Other risk factors may be operative but are poorly understood at this time.

Over a 10-year time period, Hahn et al. [35] collected prospective CP microbiologic testing and clinical data on 10 patients with *de nov*o wheezing. Nine of these subjects exhibited an acute bronchitic illness and one had community-acquired pneumonia. All 10 met serological criteria for an acute primary (*n* = 8) or secondary (*n* = 2) CP infection. Of the nine patients with acute bronchitis and wheezing, four improved without treatment and five progressed to chronic asthma. The patient with pneumonia was treated with a traditional short course of a macrolide with resolution of pneumonic infiltrate, yet developed chronic bronchitis and CP was isolated by culture from his sputum 6 months later. This type of study has not been replicated but raises several questions. CP is well known to cause protean manifestations of acute respiratory illness; these observations suggest that CP may also be capable of causing protean manifestations of chronic respiratory conditions (e.g., asthma, chronic bronchitis and COPD, reviewed in [41]). Whereas some of the CP infected patients with *de novo* wheezing resolved their acute illness without treatment, others developed chronic sequelae; identification of underlying protective and promoting factors might help address the current asthma pandemic.

Once established, CP-associated asthma has been linked with increased severity in several studies. Cook et al. [42] first identified CP biomarkers in what they referred to as “brittle asthma” (asthma that was hard to control and more severe than average). An accumulating body of evidence supports the association of CP infection with asthma severity [11, 12, 43] and with steroid resistant asthma [44]. Multiple mechanisms support the biologic plausibility of these associations (reviewed in [45]). Exposure to cigarette smoke is an established factor tied to steroid resistance in asthma [46]. Similar to cigarette smoke, CP induces pulmonary bronchial epithelial ciliostasis [47]. Additionally CP infects alveolar macrophages and lung monocytes leading to enhanced production of TNF-α, IL-1β, IL-6 and IL-8; infects human bronchial smooth muscle cells to produce IL-6 and basic fibroblast growth factor (with potential effects on bronchial hyper reactivity and lung remodeling that have yet to be thoroughly investigated); and chronic infection exposes tissues to chlamydial heat shock protein 60 (cHSP60) and bacterial lipopolysaccharide (LPS) that have been associated with increased inflammation and asthma (reviewed in [48]). Lastly, CP-specific IgE has been demonstrated to be strongly associated with severe persistent asthma (80% of cases) [43] and other chronic respiratory illnesses in children severe enough to justify undergoing bronchoscopy [49]. Whereas exposure to recognized allergens can be mitigated, exposure to unrecognized bacterial “allergens” may result in chronic unrelenting exposures that could contribute to severity [43, 50]. It may prove difficult or even impossible to unravel exactly which mechanism(s) contribute to producing an “infectious asthma” phenotype.

In regard to the involvement of CP in asthma pathogenesis, the controversy of whether the association is causal or coincidental can be settled in two ways: (1) patients diagnosed with asthma can be treated with the aim of evaluating the effects of antibiotics in ameliorating asthma symptoms compared to untreated of placebo controls and (2) animal models can be performed to evaluate the role of CP in asthma initiation and/or exacerbation. Experimental animal inoculation studies may help to elucidate mechanisms underlying CP asthma pathogenesis. Over the past three decades, animal models of asthma have been extensively utilized to elucidate mechanisms of the disease, determine the activities of genes of interest, investigate cellular pathways and predict the safety and efficacy of various drugs being considered for asthma treatment.

Initial murine models of chlamydial lung infections were carried out in adult mice and seemed to closely represent acute human asthma. These studies utilized the mouse pneumonitis biovar of *C. trachomatis* (MoPn) since it is well known as a natural mouse pathogen [51] and would therefore represent the best choice for investigating host-pathogen interactions in this context. These early studies recorded extensive lung consolidation after 7 days of airway infection and found significant airway inflammation characterized by neutrophil infiltration in airway exudates [52]. These early studies also confirmed that multiple reinfections were required to induce symptoms of chronic asthma and that a Th1 immune response contributing IFN-γ and subsequently activated macrophages was necessary to clear the infection [53, 54].

More recently, many studies have utilized neonatal mouse models for infectious asthma since early studies demonstrate that neonatal T cell immune responses in both mice and human are skewed toward a Th2 cellular phenotype as a result of placental immune pressure. These Th2 cells are much less effective in the immune response compared to their adult counterparts [55, 56]. Horvat, et al. later demonstrated that neonatal chlamydial lung infection induced mixed T-cell responses that drive allergic airway disease (AAD) using a BALB/c mouse model with ovalbumin to induce AAD [57]. Further work from this group confirmed that chlamydial infection in neonatal and infant, but not adult mice, exacerbated the development of hallmark features of asthma in ovalbumin-induced allergic airways disease models. Some of these notable features include increased mucus-secreting cell numbers, IL-13 expression, and airway hyperresponsiveness [58]. Studies from our own lab confirm that early-life chlamydial airway infection induces a Th2 immune response, both airway eosinophilia and neutrophilia, and permanent alteration of lung structure and function with concomitant enhancement of the severity of allergic airways disease in later life [59]. We confirmed that neonatally infected mice never cleared the infection, showed dissemination to the liver and spleen through the peripheral circulation, and the development of *Chlamydia*-specific IgE antibodies in the infected neonates but not adult controls [59]. Recently, Hansbro et al. completed work using a bone marrow chimera reconstitution that clearly demonstrated that infant lung infection results in lasting alterations in hematopoietic cells, leading to increased severity of AAD later in adult life [60]. A significant study by Kaiko et al. [61], demonstrated that infection of bone marrow-derived dentritic cells (BMDC) promoted Th2 immunity and airways hyperreactivity in a mouse model. Intratracheal passive transfer of infected BMDC but not uninfected control BMDC into naïve Balb/c mice resulted in increased IL-10 and IL-13 in the BAL fluid [61]. These animals also showed significant increases in airways resistance and a reduction in airways compliance compared to their uninfected counterparts. These are hallmarks of asthma and further confirm the role of chlamydial infection in asthma initiation and pathology, at least in mice. A further set of experiments by Schröder et al. [62] demonstrated that adoptive transfer of lung dendritic cells from CP infected mice, but not from uninfected mice, produced eosinophilic airway inflammation after challenge with an exogenous allergen (human serum albumin) that was dose-, timing-, and MyD88-dependent. Taken together, these findings suggest it is plausible that CP infection solely of lung dendritic cells may be sufficient to induce an asthma “phenotype” that may demonstrate characteristics that are both “infectious” and “allergic”.

**Fig. 1:**
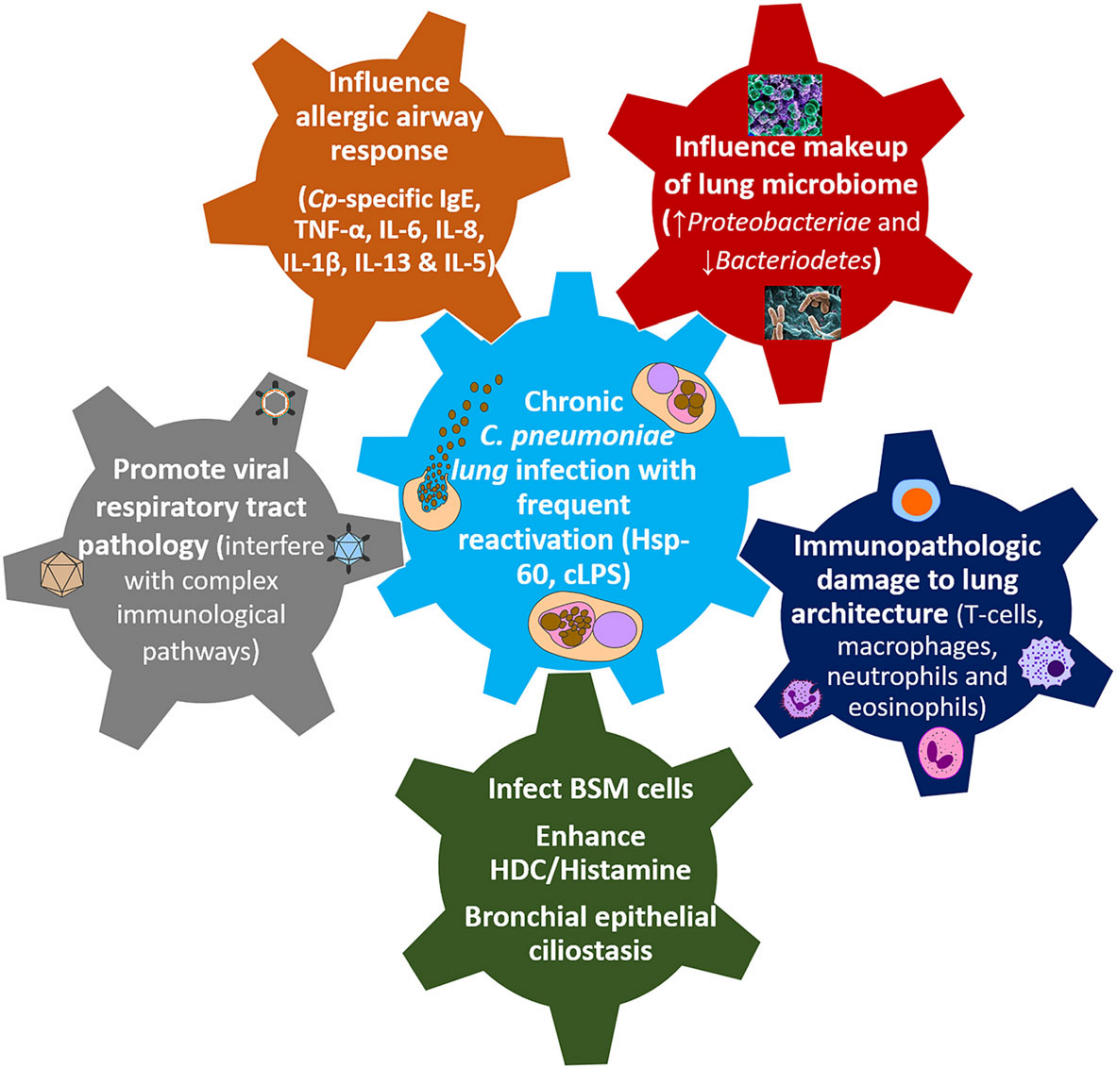
Illustration of role of chronic CP intracellular infection in asthma pathogenesis. The figure illustrates multiple pathways whereby chronic intracellular CP infection (1) is directly responsible for Immunopathologic damage and/or (2) indirectly influences allergic response as demonstrated in multiple animal models. Effects on the clinical manifestations of viral infections and the microbiome as they relate to asthma are speculative at this time. CP infection has also been shown to enhance histidine decarboxylase (HDC) to produce histamine as shown in cell culture, and the production of Cp-specific IgE antibodies is demonstrated in human asthma patients. Finally, CP infection of the airways (i) may induce hyperresponsiveness through infection of bronchial smooth muscle cells, (ii) produces inflammatory cytokines and (iii) induces ciliostasis of bronchial epithelial cells similar to the effects of cigarette smoking

These animal model studies have added significantly to our understanding of the mechanisms involved in the inflammatory process of chlamydial infection leading to asthma initiation and exacerbation. It also appears that the damage caused by chlamydial airway infections over time leads to an exaggerated airway repair or airway wall remodeling. The major features of this type of response include epithelial cell shedding, goblet cell hyperplasia, hypertrophy and hyperplasia of the airway smooth muscle bundles, basement membrane thickening and increased vascular density through angiogenesis [63]. The functional and mechanical consequences of this type of aberrant repair leads to bronchial wall thickening which can uncouple the bronchial wall from the surrounding parenchyma, significantly enhancing airway narrowing and severe obstruction [63]. This type of airway damage might prove irreversible even with long-term inhaled steroid treatment. Moreover, it is well documented that corticosteroid use drives CP out of a persistent state into active replication, since corticosteroids negatively impact several aspects of cell-mediated immunity while favoring the shift from a Th1 towards a Th2 immune response [64]. This shift in response significantly impedes the ability of the host to eradicate intracellular pathogens like CP and may lead to the release of cHSP60 which exacerbates the inflammatory process [11]. There is also evidence that CP infection may promote airway remodeling by decreasing the ratio of MMP9 to TIMP1 secreted by inflammatory cells, and by altering cellular responsiveness to corticosteroids [65]. See Fig. 1 for a summary of established and suspected mechanisms whereby CP infection may contribute to asthma pathogenesis.

## Asthma subtypes and infection

The concept that asthma is a syndrome with different underlying etiologies is well accepted. The use of the word “phenotype” to describe asthma subtypes based primarily on the inflammatory composition of respiratory secretions and/or peripheral blood is more problematic. The original definition of “phenotype” referred to relatively stable somatic manifestations of underlying genetics (such as eye color) whereas current asthma inflammatory “phenotyping” is based on cross sectional sampling of a dynamic physiologic process (host inflammatory response) and does not account for the fact that inflammatory composition is not necessary a fixed characteristic [66]. In the context of a review that focuses on chlamydial infection we are reluctant to place too much emphasis on asthma phenotypes based on inflammatory cell compositions because well described host responses to acute, sub-acute and chronic chlamydial infections involve a wide array of inflammatory cells (including eosinophils, neutrophils and monocytes) the composition of which varies significantly at different temporal stages of the infection [67]. We have commented on some fairly well defined asthma categories but even these can change over time (e.g., mild asthma can become severe, stable asthma can become uncontrolled). The dynamic and often unpredictable nature of asthma symptomatology is one of the factors that make asthma research so challenging.

### Atopic/non-atopic asthma

Historically asthma was categorized as either allergic or non-allergic but this distinction was put into question as early as the 1980s [68]. An early report of the association between CP and asthma did find independent associations of CP biomarkers, clinical allergy and asthma [40] yet in the clinical setting there is overlap between atopy and CP infection [69]. The animal models described earlier indicate that CP can promote both asthma and atopy, thus an absolute distinction between these two categories as indicators of differing underlying etiologies may not be warranted. Macrolide treatment trials that examine subgroup responses are one approach to examining the predictive value of this and other subgroups.

### Eosinophilic/neutrophilic asthma

Asthma has also been characterized as either “eosinophilic” or “neutrophilic” based on the cellular composition of respiratory secretions or bronchoalveolar lavage fluid (BALF) [70]. Simpson et al. [71] performed an RCT of a macrolide (clarithromycin) in severe refractory asthma in adults and reported no overall benefit in the group as a whole. However, there was a positive effect in the pre-specified subgroup of patients with “neutrophilic” asthma as defined by sputum IL-8 and neutrophil numbers. The predictive power of these findings is limited since it is unclear whether sputum composition is stable over time in severe refractory asthma (or any asthma, for that matter).

### Treatment

The majority of people with asthma can be well controlled with conventional guideline-based anti-inflammatory treatments (mainly inhaled steroids, sometimes in combination with an inhaled long-acting bronchodilator) [72]. Nevertheless, a significant minority of people with asthma is not well controlled by guideline treatments [73, 74]. The proportion of all people with “refractory” asthma (asthma that is not responsive to guideline therapies) has been estimated at between 5 and 15% but the contribution of refractory asthma to asthma morbidity and mortality is considerably greater, as the most severe 20% of asthma cases account for 80% of asthma morbidity and health care costs [3]. If patients with the “overlap syndrome” (asthma and COPD) are included, the numbers of people with refractory disease increases significantly [75]. Of the various novel therapies under consideration for refractory asthma [76], macrolides appear to be one of the most promising. A 2013 meta-analysis of 12 randomized, controlled trials (RCTs) of macrolides for the long term management of asthma in both adults and children found positive effects on peak expiratory flow rate (PEFR – a measure of pulmonary function), asthma symptoms, asthma quality of life (AQL), and airway hyper responsiveness (AHR), but not on forced expiratory flow rate in 1 s (FEV1) [77]. The updated 2015 Cochrane review of 18 RCTs [78] reported positive benefits on asthma symptoms and FEV1 but not on AQL (AHR and PEFR were not analyzed). A joint European Respiratory Society/American Thoracic Society (ERS/ATS) guideline on severe asthma recommends *against* the use of macrolides (“conditional recommendation, very low quality evidence”) [79]. The ERS/ATS guideline states “this recommendation places a relatively higher value on prevention of development of resistance to macrolide antibiotics, and relatively lower value on uncertain clinical benefits.” The inconsistent findings of the meta-analyses, along with the uncertainties surrounding the clinical benefits of macrolides, underscore the need for higher quality evidence. This section adds some evidence not included in the meta-analyses, reviews what is known about macrolide side effects (including the clinical consequences of resistance) and suggests research approaches to obtain better evidence. We conclude with some provisional recommendations for clinicians who may be approached by patients with new-onset, uncontrolled and/or refractory asthma who are asking for macrolide treatment.

**Fig. 2:**
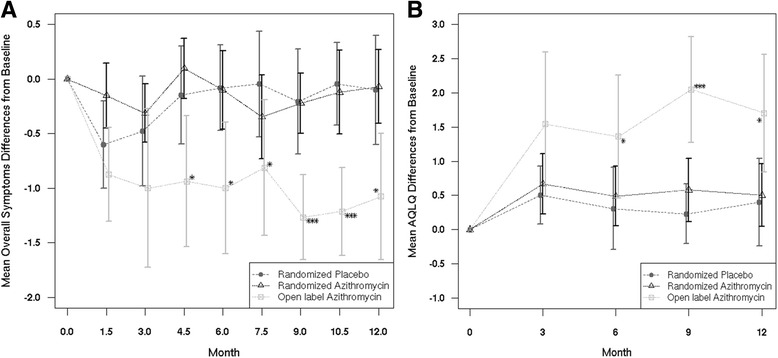
Azithromycin improves asthma symptoms and patient quality of life. Subjects with severe refractory asthma treated with azithromycin (Open Label) had fewer persisting asthma symptoms **a** and greater asthma quality of life **b** than groups with lesser asthma severity randomized to azithromycin or to placebo [44]

**Fig. 3:**
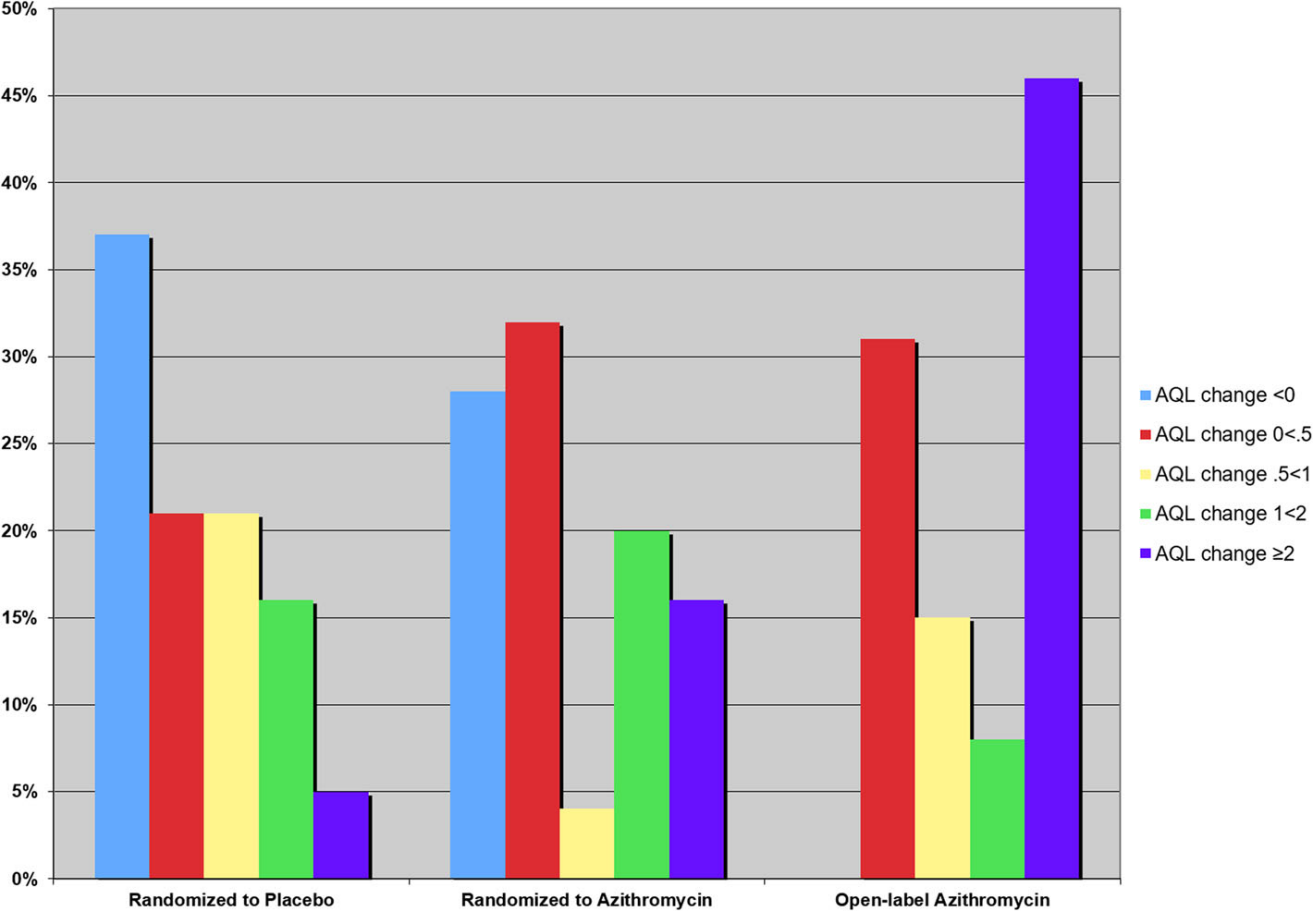
Asthma quality of life (AQL) improvement scores at 12 months (9 months after completing azithromycin). The minimum clinically important score is ≥0.5; a score of 1.5 is considered a large important change [44]

Current evidence for all asthma treatments is limited due to selection bias initiated by researchers, clinicians, and even asthma patients themselves. *Researcher bias.* The academic literature is replete with asthma efficacy studies lacking in generalizability [80]. The efficacy trials on which current asthma treatment guidelines are based systematically exclude >90% of people with asthma encountered in the general clinical population [81, 82]. Only pragmatic effectiveness trials, with minimal exclusions, are able to provide evidence applicable to the general population [44]. *Clinician bias.* A recent trial of azithromycin for acute exacerbations of asthma (AZALEA) is notable because over 95% of patients with an exacerbation were not eligible for enrollment primarily because they had received an antibiotic from a treating clinician [83]. An accompanying editorial speculated that one possible reason for the negative results of AZALEA was that clinicians were somehow able to identify and treat likely candidates, making them ineligible for the research [84]. Be that as it may, AZALEA is an example of asthma research made less informative due to non-researcher clinician behavior. *Patient bias.* Hahn et al. [44] performed a pragmatic trial of azithromycin for asthma (AZMATICS) in which the likely candidates excluded themselves from randomization. This unanticipated event occurred because AZMATICS was an Internet-based trial; people with severe, refractory asthma identified themselves as likely candidates and contacted the PI for enrollment; but upon learning that they had a 50% chance of receiving placebo, they opted out of randomization in favor of receiving a comparable azithromycin prescription from their personal clinician [85]. Rather than lose data on this “open-label” (OL) group, the study protocol was altered to include a third (OL) arm. Randomized results were similar to AZALEA (negative – see Fig. 2); however, OL subjects exhibited large and unprecedented improvements in symptoms and quality-of-life (QOL) that persisted long after treatment was completed (Figs. 2 & 3). Because the OL group was not randomized, these results do not appear in any meta-analysis of RCTs; nevertheless they strongly suggest that future macrolide RCTs should focus on the severe end of the asthma spectrum, as also recommended by others [42, 71, 86], and preferably engage patient populations that are unlikely to want to opt out of randomization.

Macrolide mechanisms of action in asthma are thought to be directly anti-inflammatory, indirectly anti-inflammatory (i.e., antimicrobial), or both. It is difficult to invoke direct anti-inflammatory macrolide effects as responsible for large clinical benefits persisting to 9 months after treatment completion. Antimicrobial effects, against specific respiratory pathogens or against the general lung microbiome, remain likely possibilities. Circumstantial evidence suggests that macrolide treatment effects may, at least in part, be attributable to antimicrobial actions against chronic atypical infections [9, 87]. This issue is by no means settled and requires further research that may be challenging given the selection biases noted above coupled with likely low sensitivity of lung sampling leading to false negative diagnosis of, for example, chronic CP lung infection [20]*.*

Azithromycin is generally well tolerated and is widely used for a variety of acute respiratory illnesses. Concerns about adverse effects of azithromycin include development of antibiotic resistance, sudden cardiac death, hearing loss and effects on the host mcrobiome. Development of resistance is a possibility whenever antibiotics are used; azithromycin is no exception. However, there are no reports of patient harm from resistant organisms in any cardiorespiratory trial performed to date [89]. Rather, the only detectable clinical effects of azithromycin in these trials were *decreased incidences* of sinusitis, acute bronchitis and pneumonia, and less use of other antibiotics [88, 89]. *Sudden cardiac death* attributable to azithromycin (1 in 4000 prescriptions in high cardiac risk patients) was plausibly documented in an epidemiologic study of a Medicaid population in Tennessee [91]. The same risk was also present for a quinolone (levofloxacin). Subsequent population-based studies in average risk populations showed no increased risk of sudden death [92, 93]. *Mild hearing loss* was reported in an excess of <1% of heart disease subjects randomized to 600 mg azithromycin *once weekly* for 12 months [88, 90]. Hearing test changes leading to discontinuation of azithromycin occurred in 2.8% of 1142 severe COPD subjects randomized to 250 mg azithromycin *daily* for 1 year [90]. The clinical significance of these hearing test changes is unclear. Notably, it is likely that daily azithromycin dosing is unnecessary [94] and may lead to increased adverse events [91]. The prolonged half-life of azithromycin within cells, including within immune system cells, allows weekly dosing and may be preferable to daily dosing when targeting either immune cells or intracellular pathogens such as CP.

Although largely speculative at this time, it appears that macrolide effects against the lung microbiome may be potentially harmful or helpful in asthma. Segal et. al. reported that an 8 week treatment with azithromycin did not alter bacterial burden but reduced α-diversity [95]. They also observed significant reduction in certain pro-inflammatory cytokines, which might explain the non-specific anti-inflammatory effects proven beneficial in COPD and asthma [95]. Published findings from Slater et al. that specifically evaluated azithromycin effects on the lung microbiome revealed a significant reduction in bacterial richness in the airway microbiota [96]. Importantly, reductions were most significant in three pathogenic genera: *Pseudomonas*, *Haemophilus* and *Staphylococcus* [96]. Overall, available data suggest that azithromycin treatment of severe asthma, while controversial, may benefit those with confirmed atypical bacterial infection [97]. Resistance, adverse events including sudden death, hearing loss and changes in host microbiome should be monitored in future pragmatic trials.

Protean manifestations of chronic CP infection, that may include asthma, chronic bronchitis, COPD, and the “overlap syndrome” (asthma and COPD) argue in favor of pragmatic trials with broad inclusion criteria that include patients with lung multi-morbidity. At least nine domains distinguish *pragmatic* (or effectiveness) trials from *explanatory* (efficacy) trials (Table 1) [98]. In the context of future RCTs of macrolides for asthma, we propose that the most important pragmatic domains are (1) broad eligibility to account for the protean clinical manifestations of both chronic reactive/obstructive lung disease and CP infections as discussed previously and (2) a comprehensive patient-centered primary outcome. Asthma exacerbations are a current popular choice as a primary outcome because they are clinically relevant [99]. However, exacerbations are only one of many outcomes that are important to asthma patients [100]. Compared to exacerbations, asthma quality-of-life (QOL) more comprehensively measures patient-important outcomes. QOL includes, but is not limited to, the adverse effects of exacerbations on patient well-being [100] and QOL has proven robust in the sole pragmatic macrolide-asthma trial performed to date [44] (Figs. 2 and 3). Many patients in this pragmatic trial [44] had significantly decreased asthma QOL at study entry and large important improvements in QOL after azithromycin, but did not experience exacerbations. This significant subgroup would have been either possibly ineligible for inclusion or not counted as successes in a trial using exacerbations as the primary outcome.

**Table 1:** Proposed design for a randomized trial of azithromycin for the long-term management of asthma. Seven of nine PRECIS-2 [98] domains are recommended as pragmatic and two as explanatory

DOMAIN	Pragmatic or Explanatory?^a^	COMMENTS
Eligibility. Who is selected to participate in the trial?	Pragmatic	Exclusions only for safety; comorbidities included.
Recruitment. How are participants recruited into the trial?	Pragmatic	Recruited from practice sites (emergency rooms, clinics).
Setting. Where is the trial being done?	Pragmatic	Performed at the practice site.
Organization. What expertise and resources are needed to deliver the intervention?	Pragmatic	No extraordinary expertize required.
Flexibility: delivery. How should the intervention be delivered?	Pragmatic	Total weekly oral dose can be administered on any schedule desired.
Flexibility: adherence. What measures are in place to make sure participants adhere to the intervention?	Explanatory	Adherence encouraged by frequent contacts by the research team and monitored by patient report and pill count.
Follow-up. How closely are the participants followed-up?	Explanatory	3-monthly study visits to collect non-routine information (e.g., spirometry, biomarkers, QOL)
Primary outcome. How relevant is it to participants?	Pragmatic	Outcome is patient-centered (see text for discussion).
Primary analysis. To what extent are all data included?	Pragmatic	Intention-to-treat.

^a^PRECIS-2 grades on a scale from 1 (extremely explanatory) to 5 (extremely pragmatic). Column 2 presents recommendations for which end of the spectrum is emphasized

Pragmatic trials primarily ask Does this treatment work? Explanatory trials primarily ask What is the mechanism? Addressing target groups/mechanisms in pragmatic trials of macrolides is desirable and possible as secondary aims by specifying a priori hypotheses coupled with subgroup analyses. We recommend studying a wide array of biomarkers using this approach. It is notable that RCTs of macrolides have been performed and/or macrolides are being recommended in the treatment of many chronic lung conditions (diffuse pan-bronchiolitis, cystic fibrosis, bronchiectasis, COPD, post-transplant bronchiolitis obliterans) [101, 102]. A planned trial will test the effectiveness of azithromycin in patients with the “overlap syndrome” (asthma-COPD) [103]. It is time to add asthma to the growing list of chronic respiratory conditions that are being evaluated by robust macrolide RCTs that are pragmatic in nature.

In the meantime, patients with severely uncontrolled and/or refractory asthma, or new-onset asthma are increasingly searching the Internet for new information and are sometimes better informed than their doctor about current evidence regarding macrolides for asthma (Hahn: personal observations). Pending more robust data from asthma RCTs that have yet to be performed, how should practicing clinicians respond when such patients request macrolide treatment? As stated above, the ERS/ATS guidelines on severe asthma recommend against the use of macrolides, albeit with caveats that the evidence for this recommendation is weak and provisional [79]. Informal guidelines from a pulmonology research group state that they recommend macrolide treatment only for confirmed diagnoses of atypical lung infection [104]. From a practical standpoint, their recommendation limits treatment only to those who have undergone bronchoscopy; even then the diagnostic sensitivity is likely to be less than perfect due to sampling issues discussed earlier. Both these recommendations have met resistance from patients who have read and understood the evidence (Hahn: personal communication). We offer a third alternative recommendation, repeated word for word from the conclusion of the sole practice-based pragmatic trial of azithromycin for asthma conducted to date [44]:

“Pending further randomized trials, given the relative safety of azithromycin and the significant disease burden from severe refractory asthma, prescribing prolonged azithromycin therapy to patients with uncontrolled asthma may be considered by managing clinicians, particularly for patients who have failed to respond to conventional treatment and as an alternative to instituting immunomodulatory agents”.

Interested clinicians and others wishing more information on patient experiences, scientific evidence and treatment alternatives are referred to a book on the subject [69].

## Conclusions

Evidence supports a complex interaction between host genetics/immune response and environmental factors (e.g., viral infections, microbiome) in the development, exacerbation and severity of asthma. Emerging evidence from animal models and human studies points to *Chlamydia pneumoniae* (CP) as a key player in this complex scenario. Future research is required to unravel the quantitative contribution of CP to asthma pathogenesis, and pragmatic treatment trials are recommended to investigate therapeutic implications.

## Abbreviations

AAD: Allergic airway disease
AHR: Airway hyper responsiveness
AOA: Adult-onset asthma
AQL: Asthma quality of life
AZALEA: Azithromycin for acute exacerbations of asthma
AZMATICS: AZithroMycin/Asthma: trial in community settings
BMDC: Bone marrow-derived dendritic cells
cHSP60: Chlamydia heat shock protein 60
COA: Childhood-onset asthma
COPD: Chronic obstructive pulmonary disease
Cp: *Chlamydia pneumoniae*
ERS/ATS: European Respiratory Society/American Thoracic Society
FEV1: Forced expiratory flow rate in 1 second
HRV: Human rhinovirus
IFN-γ: Interferon gamma
IL: Interleukin
LPS: Lipopolysaccharides
MP: *Mycoplasma pneumoniae*
OL: Open label
PCR: Polymerase chain reaction
PEF: Peak expiratory flow
PEFR: Peak expiratory flow rate
QOL: Quality-of-life
RCTs: Randomized control trials
RSV: Respiratory syncytial virus
TNF-α: Tumor necrosis factor alpha
